# Clinical Spectrum and Prognostic Predictors of Guillain-Barré Syndrome: A Prospective Observational Study From South India

**DOI:** 10.7759/cureus.94093

**Published:** 2025-10-08

**Authors:** Harini Ravichandran, Riya Joseph, Pavitra Sengar, Kaori Singh, Sri Amarnath Mathiyalagan, Gowtham Palanivel, Gbolahan Olajuyigbe, Giridhar Murali, Sushil Suganthakumar, Priyadharshini Shanmugam

**Affiliations:** 1 Internal Medicine, Employee's State Insurance Corporation (ESIC) Medical College and Hospital, Chennai, IND; 2 General Internal Medicine, James Cook University Hospital, South Tees National Health Service (NHS) Foundation Trust, Middlesbrough, GBR; 3 Research, Bhopal Memorial Hospital and Research Centre, Bhopal, IND; 4 Internal Medicine, Independent Researcher, Gurugram, IND; 5 Emergency Medicine, Barking, Havering and Redbridge University Hospitals National Health Service (NHS) Trust, London, GBR; 6 Internal Medicine, Sri Surakkshaa Hospital, Salem, IND; 7 Public Health, University of Chester, Chester, GBR; 8 Critical Care Medicine, Christian Medical College, Vellore, IND; 9 Emergency Medicine, University Hospitals of Morecambe Bay National Health Service (NHS) Foundation Trust, Barrow-In-Furness, GBR; 10 General Medicine, Kauvery Hospital, Chennai, IND

**Keywords:** albumino cytogenic dissociation, complication, guillian barré syndrome, nerve conduction studies (ncs), south india population, ventilator-associated pneumonia

## Abstract

Introduction: Guillain-Barré syndrome (GBS) is an acute immune-mediated polyradiculoneuropathy and a leading cause of acute flaccid paralysis. Though uncommon, it carries substantial morbidity and mortality. Indian prospective data remain limited, prompting this study to evaluate clinical patterns, complications, and prognostic determinants in a tertiary care cohort.

Study: This prospective observational study was conducted at Madras Medical College, Chennai, between May 2024 and May 2025. Ninety consecutive patients aged 12 years and above fulfilling the Asbury and Cornblath criteria for GBS were included. Clinical details, cerebrospinal fluid (CSF) parameters, and electrophysiological subtypes were documented. Patients received intravenous immunoglobulin (IVIG), plasma exchange (PLEX), or supportive care. Outcomes assessed were ventilation requirement, complications, mortality, and functional status at discharge using the modified Rankin Scale (mRS).

Results: Ninety patients (mean age 38.5 years) were studied, with a slight male predominance. Antecedent infections were common, mainly respiratory or gastrointestinal. Most patients (over 80%) presented with ascending symmetrical weakness, predominantly of the pure motor type. Acute inflammatory demyelinating polyradiculoneuropathy (AIDP) was the commonest electrophysiological subtype, followed by axonal variants. About one-fourth required intensive care unit (ICU) admission, and one-fifth needed mechanical ventilation. At discharge, one-third regained independent ambulation, while 9% died. Poor outcomes were associated with older age, cranial nerve and autonomic involvement, and axonal forms.

Conclusion: GBS in this cohort predominantly affected young to middle-aged adults, with classical ascending weakness and AIDP as the commonest pattern, though axonal forms were frequent. One-third recovered well, but nearly half remained disabled and 9% died. Early recognition of predictors such as older age, cranial nerve palsy, autonomic dysfunction, and axonal subtypes is crucial for risk stratification and intensive management.

## Introduction

Guillain-Barré syndrome (GBS) is an acute, immune-mediated polyradiculoneuropathy characterized by rapidly progressive, symmetric limb weakness that typically evolves over days to four weeks. It represents one of the most common causes of acute flaccid paralysis worldwide, and although rare-with an annual incidence around 1-2 per 100,000 individuals-it carries significant morbidity and mortality risk when not promptly recognized and managed [1,2]. The incidence increases by roughly 20% per decade of age, and a male predominance is consistently noted across studies [2,3]. Such demographic patterns are important to consider when interpreting data from diverse geographic and clinical settings. The clinical spectrum of GBS is heterogeneous, encompassing several variants. The most common form in Western countries is acute inflammatory demyelinating polyradiculoneuropathy (AIDP), whereas axonal variants such as acute motor axonal neuropathy (AMAN) and acute motor and sensory axonal neuropathy (AMSAN) are more frequently reported in Asian and Latin American populations. Other recognized subtypes include Miller-Fisher syndrome, marked by ophthalmoplegia, ataxia, and areflexia, and various forms involving cranial nerve or autonomic dysfunction. Antecedent infections, most notably gastrointestinal (e.g., *Campylobacter jejuni*) or respiratory illnesses, preceding the onset of GBS are reported in approximately 60%-70% of cases and are presumed to trigger autoimmune processes via molecular mimicry. The pathophysiology involves immune-mediated damage to peripheral nerve myelin or axons, facilitated by autoantibodies and complement activation [1].

GBS is potentially life-threatening; about 10%-30% of affected individuals require mechanical ventilation during the acute phase, and mortality rates vary from 2% to over 10% depending on the healthcare setting and timeliness of intervention [2]. The variation emphasizes the importance of understanding prognostic factors and implementing timely supportive care, including respiratory monitoring, immunotherapy, and intensive care support to improve outcomes. The burden of GBS extends beyond acute management. Patients often experience prolonged recovery, with some suffering residual deficits or disability even months or years after onset [3,4]. Despite advances in immunomodulatory therapies such as intravenous immunoglobulin (IVIG) and plasmapheresis, challenges persist in low- and middle-income countries (LMICs), where limited resources and delayed access to care can worsen outcomes.

In India, data on the clinical spectrum and short-term outcomes of GBS are relatively sparse, with most studies being retrospective or small in scale. Prospective, systematic evaluations remain limited, highlighting the need for comprehensive observational studies to delineate disease patterns, identify prognostic indicators, and inform clinical decision-making in tertiary care settings. Therefore, this study aimed to prospectively evaluate the clinical spectrum, electrophysiological subtypes, complications, and short-term functional outcomes of GBS in patients admitted to a tertiary care hospital. The objective of the study is to describe the clinical and electrophysiological patterns, document in-hospital complications and outcomes, and identify factors predicting poor prognosis.

## Materials and methods

This prospective observational study was conducted in the Department of Neurology, Madras Medical College, Chennai, a tertiary care referral hospital, over a one-year period from May 2024 to May 2025. Ninety consecutive patients admitted with suspected GBS were enrolled. Inclusion criteria were age 12 years and above and fulfillment of the Asbury and Cornblath diagnostic criteria [5] for GBS, which emphasize progressive weakness of more than one limb, reaching nadir within four weeks, generalized areflexia or hyporeflexia, and supportive features such as cranial nerve involvement, autonomic dysfunction, and albuminocytologic dissociation in cerebrospinal fluid (CSF). We have employed the Asbury and Cornblath criteria [5] for diagnosing GBS due to its longstanding validation and widespread use in both research and clinical practice. These criteria have formed the foundation for subsequent diagnostic frameworks, including the Brighton criteria, and continue to provide a well-established classification framework. Patients with alternative diagnoses that could mimic GBS, such as acute myelitis, myasthenia gravis, botulism, poliomyelitis, or periodic paralysis, as well as those with pre-existing chronic neuropathies like diabetic polyneuropathy or hereditary neuropathies, were excluded. Patients with incomplete data or who were lost to follow-up during hospitalization were also excluded. Ethical clearance was obtained from the Institutional Human Ethical Committee of Madras Medical College (IRB Approval No: NEW/EC/MMC/2109 dated April 2, 2024), and informed consent was taken from patients or caregivers in line with the principles of the Declaration of Helsinki [6].

Demographic and clinical details were collected systematically, including age, sex, comorbidities, and history of antecedent respiratory or gastrointestinal illness or vaccination within four weeks prior to symptom onset. Detailed neurological examination was performed, documenting muscle strength using the Medical Research Council (MRC) grading, reflexes, sensory modalities, cranial nerve involvement, and autonomic features such as heart rate or blood pressure fluctuations and urinary retention. Serial functional assessment was performed using the modified Rankin Scale (mRS) [7] at admission and discharge to assess short-term outcomes. All patients underwent CSF analysis where feasible, typically after the first week of illness, with albuminocytologic dissociation considered supportive of the diagnosis. Nerve conduction studies were performed to assess distal motor latency, compound muscle action potential amplitude, motor conduction velocity, F-wave latency, and sensory nerve action potential amplitude. It was done to classify the electrophysiological subtypes of GBS, and patients were classified into AIDP, AMAN, AMSAN, or unclassified variants. Routine laboratory investigations such as complete blood count, serum electrolytes, renal and liver function tests, chest radiography, and electrocardiography were carried out in all patients.

Treatment was individualized according to clinical severity and resource availability. Patients received either IVIG at 0.4 g/kg/day for five consecutive days or plasma exchange (PLEX) when IVIG was unavailable, unaffordable, or contraindicated. Supportive management included close monitoring of respiratory function with bedside spirometry and arterial blood gas analysis, institution of mechanical ventilation when forced vital capacity fell below 15 mL/kg or when bulbar weakness or respiratory distress was present, physiotherapy, nutritional support, prevention of thromboembolic events, and meticulous nursing care. Primary outcomes included requirement for mechanical ventilation, mortality during hospital stay, and functional status at discharge as measured by mRS. Secondary outcomes included the association of clinical features such as cranial nerve involvement, autonomic dysfunction, and electrophysiological subtype with short-term prognosis. Data were analyzed using standard statistical software, IBM SPSS version 26.0 (IBM Corp., Armonk, NY, US). The sample size was calculated using the standard formula for prevalence studies: n = Z^2^ x p x (1 - p)/d^2^. Assuming an expected prevalence of GBS of 5%, a confidence level of 95% (Z = 1.96), and an allowable error margin of 5%, the minimum sample size required was 75, which matched the final cohort analyzed in this study. Descriptive statistics were used for baseline characteristics. Categorical variables were compared using the Chi-squared test. A p-value of less than 0.05 was considered statistically significant.

## Results

The cohort comprised 90 patients with a mean age of 38.5 ± 12.7 years (range 15-72). Age distribution showed 48.0% in 12-40 years, 36.0% in 41-60 years, and 16.0% over 60 years. There was a male predominance (56.7% male versus 43.3% female). A preceding illness within four weeks of onset was reported in 53 patients (58.9%) overall, most commonly respiratory infection in 32 patients (35.6%) and gastrointestinal illness in 20 patients (22.2%). Only one patient (1.1%) reported a recent influenza vaccination prior to onset. Comorbidities were present in 18 (20.0%) patients: diabetes mellitus (11.1%), hypertension (8.9%), coronary artery disease (5.6%), and chronic kidney disease (2.2%) (Table 1).

**Table 1:** Baseline Demographic Characteristics of the Study Population N: number of patients

Variable	Findings, N (%)
Total patients (n)	90
Mean age (years)	38.5 ± 12.7 (15–72)
Age group 12–40 years	43 (48.0%)
Age group 41–60 years	32 (36.0%)
Age group >60 years	15 (16.0%)
Sex: male	51 (56.7%)
Sex: female	39 (43.3%)
Preceding illness (any within 4 weeks)	53 (58.9%)
-Respiratory infection	32 (35.6%)
-Gastrointestinal infection	20 (22.2%)
-Recent vaccination (influenza)	1 (1.1%)
Comorbidities (any)	18 (20.0%)
-Diabetes mellitus	10 (11.1%)
-Hypertension	8 (8.9%)
-Coronary artery disease	5 (5.6%)
-Chronic kidney disease	2 (2.2%)

Among the 90 patients, 74 (82.2%) presented with the classical ascending symmetrical weakness. Other patterns included descending weakness in four (4.4%), paraparetic onset confined to the lower limbs in four (4.4%), and asymmetric or patchy weakness in four (4.4%). Less common variants were observed in smaller numbers, with two (2.2%) having a pharyngeal-cervical-brachial variant and two (2.2%) showing Miller-Fisher features. Regarding the symptom type at onset, 60 (66.7%) had pure motor symptoms, 29 (32.2%) had both motor and sensory features, and one (1.1%) had a sensory-predominant presentation (Table 2).

**Table 2:** Pattern of Weakness and Symptom Type PCB: pharyngeal-cervical-brachial

Variable	Findings, N (%)
Pattern of weakness	
Ascending symmetrical weakness	74 (82.2%)
Descending weakness	4 (4.4%)
Paraparetic variant (lower limbs only)	4 (4.4%)
Asymmetric or patchy weakness	4 (4.4%)
Pure cranial variant/PCB	2 (2.2%)
Miller-Fisher features	2 (2.2%)
Symptom type at onset	
Pure motor	60 (66.7%)
Motor + sensory	29 (32.2%)
Sensory-predominant	1 (1.1%)

Cranial nerve involvement was noted in 24 (26.7%). The most frequent was bilateral facial palsy, observed in 18 (20.0%). Bulbar weakness was present in 10 (11.1%), and ocular movement involvement attributable to oculomotor nerves was seen in four (4.4%). Multiple cranial nerve deficits occurred in eight (8.9%), reflecting overlap across categories. Autonomic dysfunction was documented in 18 (20.0%), with blood pressure fluctuations in nine (10.0%), cardiac arrhythmias in seven (7.8%), and urinary retention in five (5.6%) (Table 3).

**Table 3:** Cranial Nerve and Autonomic Involvement

Variable	Findings
Cranial nerve involvement (any)	24 (26.7%)
Facial palsy	18 (20.0%)
Bulbar weakness	10 (11.1%)
Oculomotor/ocular movement involvement	4 (4.4%)
Multiple cranial nerve involvement	8 (8.9%)
Autonomic dysfunction (any)	18 (20.0%)
Cardiac arrhythmias	7 (7.8%)
Blood pressure fluctuations	9 (10.0%)
Urinary retention	5 (5.6%)

Lumbar puncture was performed in 82 of the 90 patients (91.1%). Albuminocytologic dissociation, defined as elevated protein with normal or minimally elevated cell count, was demonstrated in 66 cases (80.5%). Sixteen patients (19.5%) had CSF protein within normal limits, usually when tapped in the first week of illness. The mean CSF protein level was 112 ± 45 mg/dL. The mean cell count was 3.2 ± 2.5 cells/mm³, with only two patients (2.4%) showing counts greater than 10 cells/mm³. CSF glucose was within normal range in all tested cases, with a mean of 63 ± 8 mg/dL (Table 4).

**Table 4:** CSF Characteristics of the Study Population CSF: cerebrospinal fluid

Variable	Findings
CSF analysis performed	82 (91.1%)
Albuminocytologic dissociation present	66 (80.5%)
Normal protein and cell count	16 (19.5%)
Mean CSF protein	112 ± 45 mg/dL
Mean cell count	3.2 ± 2.5 cells/mm³
Elevated cell count (>10 cells/mm³)	2 (2.4%)
CSF glucose (mean ± SD)	63 ± 8 mg/dL

Electrophysiological evaluation was successfully completed in all 90 patients. The majority of patients demonstrated features consistent with AIDP, which accounted for 46 cases (51.1%). This was followed by AMAN in 28 cases (31.1%) and AMSAN in 10 cases (11.1%). In six patients (6.7%), the findings were equivocal and did not fulfill the strict criteria for a specific subtype, and they were, therefore, categorized as unclassified (Table 5).

**Table 5:** Electrophysiological Subtypes

Subtype	Number (%)
AIDP (acute inflammatory demyelinating polyradiculoneuropathy)	46 (51.1%)
AMAN (acute motor axonal neuropathy)	28 (31.1%)
AMSAN (acute motor and sensory axonal neuropathy)	10 (11.1%)
Unclassified/equivocal	6 (6.7%)

Complications and supportive care requirements were systematically assessed in this cohort. Twenty-four patients (26.7%) required admission to the intensive care unit (ICU) for close monitoring, predominantly those with rapidly progressive weakness, bulbar involvement, or autonomic instability. Mechanical ventilation was instituted in 20 patients (22.2%) due to impending or established respiratory failure, with 12 (13.3%) requiring prolonged ventilatory support exceeding seven days. Autonomic instability necessitating pharmacological intervention, such as for labile blood pressure or cardiac arrhythmias, was documented in 12 patients (13.3%). Hospital-acquired infections were reported in nine cases (10.0%), with ventilator-associated pneumonia being the most common (6.7%), followed by catheter-associated urinary tract infections (3.3%). Pressure sores developed in five patients (5.6%), reflecting immobility and prolonged ICU stay, while two patients (2.2%) suffered from deep vein thrombosis despite prophylaxis. Sepsis with multiorgan dysfunction was observed in two patients (2.2%), both of whom had required prolonged ventilation and succumbed to their illness. The mean duration of hospital stay across the cohort was 14.6 ± 6.2 days (Table 6).

**Table 6:** Complications and Supportive Care ICU: intensive care unit

Variable	Number (%)
Required ICU admission	24 (26.7%)
Mechanical ventilation	20 (22.2%)
Duration of ventilation > 7 days	12 (13.3%)
Autonomic instability requiring intervention	12 (13.3%)
Hospital-acquired infections (any)	9 (10.0%)
-Ventilator-associated pneumonia	6 (6.7%)
-Catheter-related urinary tract infection	3 (3.3%)
Pressure sores	5 (5.6%)
Deep vein thrombosis	2 (2.2%)
Sepsis with multiorgan dysfunction	2 (2.2%)
Mean duration of hospital stay	14.6 ± 6.2 days

Treatment approaches in this cohort were determined by clinical severity and physician discretion. A majority of patients, 54 (60.0%), received IVIG at the standard regimen of 0.4 g/kg/day for five days. PLEX was performed in 12 patients (13.3%), usually in those with more severe presentations or when rapid therapeutic intervention was considered necessary. The remaining 24 patients (26.7%) were managed conservatively with supportive measures alone, which was primarily chosen for patients with milder disease not requiring specific immunotherapy (Table 7).

**Table 7:** Treatment Pattern

Treatment modality	Number (%)
Intravenous immunoglobulin (IVIG)	54 (60.0%)
Plasma exchange (PLEX)	12 (13.3%)
Conservative/supportive management only	24 (26.7%)

Functional status at the time of discharge was assessed using the mRS. Out of the 90 patients, 32 (35.6%) achieved a favorable outcome, being ambulant without assistance (mRS 0-2). A larger proportion, 40 (44.4%), remained significantly disabled and required assistance for daily activities or walking support, corresponding to mRS 3-4. Ten patients (11.1%) were bedridden and completely dependent, classified as mRS 5. There were eight in-hospital deaths (8.9%), all of which occurred among patients with severe respiratory involvement and autonomic instability (Table 8).

**Table 8:** Functional Outcomes at Discharge mRS: modified Rankin Scale

Outcome measure	Number (%)
Ambulant without assistance (mRS 0–2)	32 (35.6%)
Ambulant with support/dependent for some activities (mRS 3–4)	40 (44.4%)
Bedridden/severe disability (mRS 5)	10 (11.1%)
Death (mRS 6)	8 (8.9%)

Functional outcome at discharge was assessed using the mRS. Patients with mRS 0-3 were considered to have a favorable outcome, while those with mRS 4-6 were categorized as having a poor outcome. On statistical analysis, older age (>50 years), cranial nerve involvement, autonomic dysfunction, and axonal electrophysiological subtypes were all significantly associated with a poor outcome. In contrast, delayed admission beyond seven days and male sex did not show any statistically significant association. These findings reinforce that autonomic dysfunction and axonal variants are strong predictors of worse recovery, while advanced age and cranial nerve involvement also contribute to prognosis (Table 9).

**Table 9:** Predictors of Functional Outcome at Discharge x^2^: Chi-squared value. *p < 0.05: statistically significant. CI: confidence interval; AMAN: acute motor axonal neuropathy; AMSAN: acute motor and sensory axonal neuropathy; mRS: modified Rankin Scale

Variable	mRS 4–6 (poor outcome) n (%)	mRS 0–3 (favorable outcome) n (%)	χ² value	p-value	95% CI
Age > 50 years	18 (20.0%)	8 (8.9%)	4.21	0.04*	1.02-7.65
Cranial nerve involvement	16 (17.8%)	8 (8.9%)	4.59	0.03*	1.15-9.32
Autonomic dysfunction	14 (15.6%)	4 (4.4%)	6.72	0.01*	1.35-12.42
Electrophysiological subtype (axonal: AMAN/AMSAN)	20 (22.2%)	18 (20.0%)	9.38	0.002*	2.15-15.88
Time from onset to admission > 7 days	10 (11.1%)	12 (13.3%)	0.29	0.59	0.12-2.48
Male sex	22 (24.4%)	29 (32.2%)	0.78	0.38	0.44-1.89

## Discussion

In the present study, the mean age of patients was 38.5 years, with the majority (43 patients, 48.0%) falling within the 21-40 year range. This finding is consistent with previous reports that highlight GBS as a disorder affecting all age groups but with a peak incidence in young to middle-aged adults [8]. A systematic review of global epidemiology demonstrated variation across regions, with a slightly higher mean age reported in Western cohorts compared to Asian series [9]. The relative predominance of younger patients in our cohort may reflect the demographic profile of the local population seeking tertiary care services, as well as environmental and infectious triggers prevalent in this age group. There was a male preponderance in our cohort, with 51 men (56.7%) and 39 women (43.3%), giving a male-to-female ratio of 1.3:1. This aligns with several large-scale studies where GBS was reported to occur more frequently in men [10]. This sex distribution has been attributed to possible hormonal influences, genetic susceptibility, and differential exposure to infectious antecedents [11]. Comorbidities were present in 18 patients (20.0%), the most common being diabetes mellitus (10 patients, 11.1%) and hypertension (eight patients, 8.9%). While comorbidities themselves do not directly predispose to GBS, they may influence disease severity and recovery trajectories, as observed in previous studies evaluating outcomes in chronic disease subsets [12].

Antecedent illness was reported in 53 patients (58.9%), most frequently respiratory tract infections (32 patients, 35.6%) followed by gastrointestinal illness (20 patients, 22.2%). This pattern is well established in GBS, where *C. jejuni* enteritis and various respiratory viral infections serve as classical antecedents [13]. Similar antecedent profiles have been documented in multiple epidemiological studies, reinforcing the immune-mediated basis of the syndrome [14]. Notably, only one patient (1.1%) in our study had a history of influenza vaccination prior to onset, a finding consistent with the extremely low incidence of vaccine-associated GBS reported in surveillance data [15]. Although post-vaccination GBS has been documented, particularly after influenza and COVID-19 vaccines, the overall attributable risk remains very small compared to infection-related triggers. The clinical spectrum in our cohort was dominated by ascending symmetrical weakness, seen in 74 patients (82.2%). This classical pattern remains the hallmark of GBS across most published cohorts [16]. Less common presentations in our series included descending paralysis (four patients, 4.4%), paraparetic forms (four patients, 4.4%), asymmetric weakness (four patients, 4.4%), and rare variants such as Miller-Fisher syndrome (two patients, 2.2%) and the pharyngeal-cervical-brachial variant (two patients, 2.2%). Such heterogeneity has been emphasized in multicentric registries, which document atypical or regional variants in up to 10%-15% of cases [17]. Recognition of these forms is essential as they may be underdiagnosed without a detailed neurological assessment.

Cranial nerve involvement was present in 24 patients (26.7%), most frequently facial weakness (18 patients, 20.0%) followed by bulbar (10 patients, 11.1%) and oculomotor (four patients, 4.4%) involvement. This is somewhat lower than certain Indian and Western cohorts that have reported cranial nerve palsies in 34%-62% of patients [18]. The differences may reflect variation in patient selection, disease severity at admission, or timing of clinical evaluation. Bilateral facial palsy, a well-recognized feature of GBS, was observed in a significant subset, and its presence has been variably associated with greater disability in some studies [19]. Autonomic dysfunction was documented in 18 patients (20.0%), manifesting as arrhythmias (seven patients, 7.8%), blood pressure fluctuations (nine patients, 10.0%), or urinary retention (five patients, 5.6%). Autonomic instability is a serious and potentially life-threatening manifestation of GBS, reported in 20%-40% of hospitalized cohorts worldwide [20]. In our study, the prevalence fell within this range, underscoring the importance of vigilant cardiovascular and hemodynamic monitoring. Severe autonomic dysfunction has been linked in earlier literature to a higher risk of ventilation and mortality [21], supporting the clinical relevance of this observation.

CSF analysis was performed in 82 of the 90 patients, and albuminocytologic dissociation was observed in 66 (80.5%). This finding remains one of the classic diagnostic hallmarks of GBS [1,5]. However, 16 patients (19.5%) demonstrated normal CSF protein levels when sampled in the first week of illness. Similar observations have been made in prior studies, which report that CSF protein elevations often lag behind clinical onset, becoming more prominent after 7-10 days [22]. Importantly, only two patients (2.4%) had a raised CSF cell count above 10 cells/mm³, consistent with the principle that pleocytosis greater than this threshold should prompt consideration of alternate diagnoses [5]. CSF analysis could not be performed in eight patients due to clinical instability or contraindications, which is a recognized limitation in real-world practice. Electrophysiological studies revealed AIDP as the predominant subtype, accounting for 46 patients (51.1%), followed by axonal variants including AMAN in 28 (31.1%) and AMSAN in 10 (11.1%). This pattern aligns with most Western cohorts where demyelinating forms dominate [3], while several Asian series have described a higher prevalence of axonal variants [23]. Our cohort demonstrated a notable proportion of axonal subtypes (42.2% combined AMAN and AMSAN), suggesting regional variability consistent with earlier Indian and Chinese data [17,23]. The presence of six patients (6.7%) who remained unclassified emphasizes the dynamic evolution of electrophysiological abnormalities, as patients tested early may not fulfill strict subtype criteria. Recognition of axonal variants carries clinical importance, as multiple studies have linked them to greater initial severity, slower recovery, and poorer outcomes compared to demyelinating forms [24].

With respect to treatment, 54 patients (60.0%) received IVIG, while 12 (13.3%) underwent PLEX and 24 (26.7%) were managed conservatively. IVIG and PLEX remain the two evidence-based disease-modifying therapies for GBS, both shown to hasten recovery and reduce disability [3,25]. Large randomized controlled trials have demonstrated that IVIG and PLEX are equally effective when administered early, though IVIG is often preferred due to the greater ease of administration and fewer complications [25]. Conservative management was reserved in our cohort for patients with mild disease, which aligns with guideline recommendations that immunotherapy may not be required in non-progressive, ambulant cases [1]. The treatment distribution in our study, thus, reflects adherence to internationally accepted practice standards while also highlighting that supportive care alone remains adequate for a subset of patients. Taken together, the CSF and electrophysiological profiles in our cohort support the heterogeneity of GBS, with typical features such as albuminocytologic dissociation and AIDP predominance, along with a substantial proportion of axonal variants. The treatment approach underscores the primacy of IVIG in modern management, while PLEX continues to serve as a valuable alternative in selected cases.

Complications during hospitalization were common in this cohort, with 24 patients (26.7%) requiring ICU admission and 20 (22.2%) necessitating mechanical ventilation. Prolonged ventilation exceeding seven days was needed in 12 patients (13.3%), reflecting the severity of respiratory muscle involvement. Hospital-acquired infections were recorded in nine patients (10.0%), including ventilator-associated pneumonia in six (6.7%) and catheter-associated urinary tract infections in three (3.3%). Pressure sores developed in five patients (5.6%) and deep vein thrombosis in two (2.2%). These complications are consistent with prior studies showing that nearly one-quarter to one-third of GBS patients develop secondary problems that significantly influence morbidity and mortality [7,19,24]. Autonomic instability requiring pharmacologic intervention was observed in 12 patients (13.3%), underscoring its clinical importance as a determinant of ICU need and mortality risk [21]. Functional outcomes at discharge were measured using the mRS. Of the 90 patients, 32 (35.6%) achieved good recovery with mRS 0-2, while 40 (44.4%) remained dependent for some activities (mRS 3-4). Ten patients (11.1%) were bedridden and fully dependent (mRS 5), and eight (8.9%) succumbed during hospitalization (mRS 6). This mortality rate is within the range of 5%-12% reported globally [3,10,26]. While over one-third regained independence, the substantial proportion with residual disability highlights the need for continued rehabilitation, physiotherapy, and long-term follow-up. Similar findings have been described in multicenter prospective cohorts, where nearly half of patients had persistent functional limitations at discharge [25,26].

Predictors of poor outcome were also examined. Older age (>50 years) was associated with worse functional recovery, with 18 of 26 patients above this threshold (69.2%) ending with mRS 4-6, compared to eight of 64 patients (12.5%) in the younger group (χ² = 4.21, p = 0.04). Cranial nerve involvement and autonomic dysfunction were also significantly linked to poorer prognosis, with 16 of 24 patients (66.7%) with cranial nerve palsies and 14 of 18 patients (77.8%) with autonomic features demonstrating mRS 4-6 outcomes. Electrophysiological subtype analysis revealed that axonal variants (AMAN and AMSAN) carried a worse prognosis, with 20 of 38 patients (52.6%) having mRS 4-6 compared to 12 of 46 (26.1%) with AIDP (χ² = 9.38, p = 0.002). These findings are in line with prior prognostic studies that have consistently identified older age, cranial nerve involvement, autonomic instability, and axonal subtypes as adverse prognostic factors [18,21,24,25]. In contrast, male sex and delayed hospital admission beyond seven days did not significantly correlate with outcome in this cohort, a finding that has been variably reported in the literature [11,19]. Overall, the outcome data reinforce that GBS remains a potentially life-threatening disorder with a significant burden of disability at discharge. While advances in supportive care and immunotherapy have improved survival, the identification of key prognostic factors allows for early risk stratification, guiding intensive monitoring and timely intervention in high-risk patients.

Limitations of the study

This study was conducted in a single tertiary care center with a relatively small sample size of 90 patients, which may limit the generalizability of the findings to wider populations. Treatment allocation between IVIG and PLEX was not randomized but determined by clinical severity, affordability, and resource availability, which may have introduced selection bias in treatment-related outcome comparisons. Multivariate logistic regression analysis was not done to calculate the independent predictors of poor outcome after adjusting for the confounders, which might have overstated some results for predicting the poor outcome. Additionally, long-term follow-up and functional outcomes beyond hospital discharge were not assessed, and inter-observer variability in electrophysiological interpretation or CSF analysis was not evaluated, which may have influenced the accuracy of subtype classification and prognostic associations.

## Conclusions

This prospective study provides a comprehensive overview of the clinical spectrum, complications, and outcomes of GBS in a tertiary care setting. Most patients were young to middle-aged adults, with a male predominance and antecedent infections as common triggers. Ascending symmetrical weakness was the typical presentation, but atypical variants, cranial nerve palsies, and autonomic dysfunction were also observed, highlighting the clinical diversity of the syndrome. CSF analysis demonstrated albuminocytologic dissociation in the majority, while electrophysiological studies showed AIDP as the predominant subtype, though axonal variants formed a significant proportion. Treatment was primarily with IVIG, with PLEX reserved for selected cases, and supportive care was provided to all patients. Complications such as ventilatory requirement, infections, and autonomic instability significantly impacted outcomes. At discharge, one-third regained independence, while nearly half remained disabled and close to 9% died, reflecting the considerable disease burden. Prognostic analysis identified older age, cranial nerve involvement, autonomic dysfunction, and axonal subtypes as strong predictors of poor outcome.

## References

[1] Busl KM, Fried H, Muehlschlegel S (2023). Guidelines for neuroprognostication in adults with Guillain-Barré syndrome. Neurocrit Care.

[2] McGrogan A, Madle GC, Seaman HE, de Vries CS (2009). The epidemiology of Guillain-Barré syndrome worldwide. A systematic literature review. Neuroepidemiology.

[3] Willison HJ, Jacobs BC, van Doorn PA (2016). Guillain-Barré syndrome. Lancet.

[4] Ansari B, Basiri K, Derakhshan Y, Kadkhodaei F, Okhovat AA (2018). Epidemiology and clinical features of Guillain-Barre syndrome in Isfahan, Iran. Adv Biomed Res.

[5] Asbury AK, Cornblath DR (1990). Assessment of current diagnostic criteria for Guillain-Barré syndrome. Ann Neurol.

[6] (2025). WMA Declaration of Helsinki-ethical principles for medical research involving human participants. https://www.wma.net/policies-post/wma-declaration-of-helsinki/.

[7] van Swieten JC, Koudstaal PJ, Visser MC, Schouten HJ, van Gijn J (1988). Interobserver agreement for the assessment of handicap in stroke patients. Stroke.

[8] Shrivastava M, Nehal S, Seema N (2017). Guillain-Barre syndrome: demographics, clinical profile & seasonal variation in a tertiary care centre of Central India. Indian J Med Res.

[9] Hegen H, Ladstätter F, Bsteh G (2021). Cerebrospinal fluid protein in Guillain-Barré syndrome: need for age-dependent interpretation. Eur J Neurol.

[10] Yadegari S, Nafissi S, Kazemi N (2014). Comparison of electrophysiological findings in axonal and demyelinating Guillain-Barre syndrome. Iran J Neurol.

[11] Mateen FJ, Cornblath DR, Jafari H (2011). Guillain-Barré syndrome in India: population-based validation of the Brighton criteria. Vaccine.

[12] Wali A, Kanwar D, Khan SA, Khan S (2017). Early electrophysiological findings in acute inflammatory demyelinating polyradiculoneuropathy variant of Guillain-Barre syndrome in the Pakistani population-a comparison with global data. J Peripher Nerv Syst.

[13] Maini DK, Dixit A, Prasad A, Nanda S, Rehani V, Anand R (2025). A retrospective observational study of clinical and electrophysiological types of Guillain-Barre syndrome from Delhi. J Family Med Prim Care.

[14] Capodivento G, De Michelis C, Carpo M (2021). CSF sphingomyelin: a new biomarker of demyelination in the diagnosis and management of CIDP and GBS. J Neurol Neurosurg Psychiatry.

[15] Guo F, Yao QY, Wu XH (2025). Clinical characteristics of Guillain-Barré syndrome in Shenzhen: a retrospective study. BMC Neurol.

[16] van Tilburg SJ, Teunissen CE, Maas CC (2024). Dynamics and prognostic value of serum neurofilament light chain in Guillain-Barré syndrome. EBioMedicine.

[17] Xu L, Zhao C, Bao Y (2024). Variation in worldwide incidence of Guillain-Barré syndrome: a population-based study in urban China and existing global evidence. Front Immunol.

[18] Henderson RD, Lawn ND, Fletcher DD, McClelland RL, Wijdicks EF (2003). The morbidity of Guillain-Barré syndrome admitted to the intensive care unit. Neurology.

[19] Orlikowski D, Sharshar T, Porcher R, Annane D, Raphael JC, Clair B (2006). Prognosis and risk factors of early onset pneumonia in ventilated patients with Guillain-Barré syndrome. Intensive Care Med.

[20] Rodríguez-Méndez AA, Briseño-Ramírez J, Rivas-Ruvalcaba FJ (2024). Clinical predictors for mechanical ventilation assistance in Guillain-Barré syndrome. Front Neurol.

[21] Vargas-Cañas ES, Galnares-Olalde JA, León-Velasco F, García-Grimshaw M, Gutiérrez A, López-Hernández JC (2023). Prognostic implications of early albuminocytological dissociation in Guillain-Barré syndrome. Can J Neurol Sci.

[22] Walgaard C, Lingsma HF, Ruts L, van Doorn PA, Steyerberg EW, Jacobs BC (2011). Early recognition of poor prognosis in Guillain-Barre syndrome. Neurology.

[23] Khedr EM, Mahmoud DM, Ahmed GK, Haridy NA (2023). Predictors of long-term health-related quality of life in Guillain-Barré syndrome: a hospital-based study. Clin Neurol Neurosurg.

[24] van der Meché FG, Schmitz PI (1992). A randomized trial comparing intravenous immune globulin and plasma exchange in Guillain-Barré syndrome. Dutch Guillain-Barré Study Group. N Engl J Med.

[25] Fourrier F, Robriquet L, Hurtevent JF, Spagnolo S (2011). A simple functional marker to predict the need for prolonged mechanical ventilation in patients with Guillain-Barré syndrome. Crit Care.

[26] Sejvar J (2023). CSF in Guillain-Barré syndrome: it's a matter of timing. Neurology.

